# Acne

**Published:** 1890-03-15

**Authors:** 


### ACNE.

An abstract of a lecture delivered at the Blackfriars Hospital for Diseases of the Skin, by Dr. J. Frank Payne, has appeared in the *Lancet*. Acne punctata, a very common form of the malady, and is seen chiefly on those parts of the body which, while not absolutely free from hair, abound in rudimentary hair structures, and in sebaceous glands connected with the hair. It appears most frequently on the face, and to a slight extent on the lateral part of the neck, but never occurs on the adjacent portion of the neck above the clavicle, nor on the hairy scalp. "The parts affected are those which during the course of evolution of the human species, natural selection has succeeded in most instances in entirely depriving of their hairy investment." Treatment consists in washing with soft soap and water, friction with a towel to get the skin to act, and squeezing of the comedenes. We were under the impression that most forms of acne are best treated by internal medicines, for they as often as not depend on some dyspeptic derangement, or deteriorated state of the system. The author, however, seems to rely most on external applications.

